# Impact of corn straw and straw-derived biochar returning to the field on soil carbon fractions, carbon-converting enzyme activities, and *cbbL* bacterial community structure

**DOI:** 10.3389/fmicb.2025.1611691

**Published:** 2025-11-03

**Authors:** Jiawang Li, Qina Ren, Hang Yu, Xiangyu Wu, Yuan Yin, Zhonghui Yue, Xin Bai

**Affiliations:** Heilongjiang Province Key Laboratory of Plant Biology of General Colleges and Universities, College of Life Science and Technology, Harbin Normal University, Harbin, China

**Keywords:** corn straw, straw-derived biochar, carbon fractions, carbon-converting enzymes, soil *cbbL* bacterial community

## Abstract

**Introduction:**

Straw return is recognized as an effective practice for improving soil organic matter. However, in the black soil regions of China, limited information is available on how the individual or combined application of crop straw and straw-derived biochar influences soil carbon-converting enzymes and the soil *cbbL* bacterial.

**Methods:**

This study conducted three consecutive growing-season field experiments in a typical black-soil zone using a soybean–corn rotation system. Four straw return treatments were established based on equal carbon input (2,500 kg·hm^-2^), including the blank control with no carbon source (T0), corn straw applied alone (T1), straw-derived biochar applied alone (T2), and their co-application at ratios of 1:3 (T3) and 3:1 (T4).

**Results:**

The results indicated that compared to T0, the four treatments had no significant effect on soil labile organic carbon (LOC) but significantly effect soil organic carbon (OC), dissolved organic carbon (DOC), and microbial biomass carbon (MBC) (*p* < 0.05). Notably, soil carbon mineralization was significantly enhanced under T1 and T3, increasing by 13.38% and 13.28%, respectively. All the treatments significantly reduced the relative abundance of Alphaproteobacteria (dominant class) and *Nitrobacter* (dominant genus) in the *cbbL* bacterial community, and significantly promoted soil enzyme activities: SCL (cellulase), SAI (amylase) and SSC (sucrase) increased by 2.95–15.35%, 6.10–19.26% and 10.84–53.17%, respectively. Comprehensive analysis demonstrated that straw-derived biochar incorporation directly and significantly affected the *cbbL* bacterial community structure, while both straw and biochar significantly affected the enzyme activities. Enzyme activities directly influenced the levels of soil carbon fractions, which ultimately determined the soil organic carbon mineralization capacity.

**Discussion:**

Overall, the response of carbon mineralization to straw and biochar application was primarily driven by the content of soil carbon fractions, which were regulated by enzyme activity. This study provides a scientific basis for enhancing the carbon sequestration potential of black soils in China.

## 1 Introduction

Since the early 20th century, the greenhouse effect has emerged as a major global environmental concern, with soils being recognized as an important source of atmospheric greenhouse gasses (Roberto et al., 2015; Ahirwal et al., 2017). Among the various pathways, soil organic carbon (SOC) mineralization plays a significant role in carbon emissions from agricultural systems. This process involves the microbial decomposition of organic matter and the release of greenhouse gasses such as CO_2_ and CH_4_ into the atmosphere (Cox et al., 2000; Lal, 2004; Kan et al., 2021). Implementing appropriate agricultural management practices can help reduce the rate of SOC mineralization, thereby increasing soil carbon stocks and promoting farmland as a potential carbon sink (Lal, 2010; Chen et al., 2022). Hence, developing conservation tillage strategies to reduce SOC mineralization and enhance soil carbon sequestration has become a critical challenge for sustainable agricultural development.

Crop straw is rich in carbon, nitrogen, phosphorus, and other essential nutrients, making it a valuable resource for enhancing soil fertility (Song et al., 2018). Returning straw to the field improves the bonding strength between soil aggregates and enhances aggregate stability (Lu et al., 2013; Tian et al., 2013). This stable structure provides physical protection for internal organic carbon, reducing its accessibility to microbial decomposition and thus promoting effective SOC sequestration (Sollins et al., 1996; Pulleman and Marinissen, 2003). Therefore, straw incorporation is widely considered an effective strategy to reduce SOC mineralization and increase SOC content (Zhang et al., 2011; Zhang et al., 2014a). However, inconsistent findings have also been reported. Some studies have suggested that straw returning can stimulate SOC mineralization, thereby hindering SOC accumulation (Li et al., 2009; Zhang et al., 2015). For instance, small organic acids released during straw decomposition may solubilize mineral-bound organic matter and expose aggregate-protected carbon to microbial degradation, thereby increasing its bioavailability (Kan et al., 2020). These contradictory results may stem from variations in straw input rates (Xu et al., 2016), the duration of application (Liu et al., 2022), straw treatment methods (Li et al., 2021; Zhao et al., 2021), and soil characteristics such as texture and aggregate composition (Wu et al., 2018; Xiao et al., 2018). Moreover, excessive straw input may lead to microbial competition with crops for nutrients, organic acid accumulation, and an increase in pest and disease incidence (Li et al., 2022).

In contrast, straw-derived biochar produced via pyrolysis under oxygen-limited high-temperature conditions can mitigate these issues. It eliminates pathogens and pest residues while converting biomass into chemically stable aromatic carbon (Lehmann, 2007; Lee et al., 2019). Biochar is increasingly being recognized as a slow-release carbon source that improves soil structure, enhances fertility, and promotes crop growth (Dai et al., 2019; Han et al., 2023). Owing to its high surface area, porosity, and adsorption capacity, biochar can immobilize organic carbon, reduce microbial decomposition, and thus lower SOC mineralization, thereby effectively enhancing soil carbon storage (Kasozi et al., 2010; Hou et al., 2015; Weng et al., 2017). Some studies have reported that biochar can remain stable in the soil for thousands of years, providing a long-term carbon sequestration effect (Criscuoli et al., 2017; Vineet et al., 2024). Recent studies further indicate that the judicious application of biochar can simultaneously mitigate CH₄ and N₂O emissions and enhance soil fertility, while maintaining environmental sustainability (Ali et al., 2025). Nonetheless, the differential effects of straw and straw-derived biochar on SOC mineralization across various agroecosystems remain poorly understood and warrant further investigation.

Soil microorganisms play a vital role in regulating mineralization and carbon fixation processes within agroecosystems (Trivedi et al., 2013). Among them, autotrophic microorganisms are primarily involved in fixing atmospheric CO_2_, with ribulose-1,5-bisphosphate carboxylase/oxygenase (RubisCO) serving as the key enzyme in this process (Tabita, 1999). The *cbbL* gene encoding the large subunit of RubisCO is widely used as a molecular marker to assess the diversity of autotrophic, carbon-fixing microbial communities involved in the Calvin cycle and has been extensively applied in studies of biological carbon sequestration (Selesi et al., 2007; Yuan et al., 2013). In parallel, soil microbial communities mediate most biogeochemical cycles through the secretion of extracellular enzymes that drive the decomposition and mineralization of soil organic matter (Liang et al., 2017; Ibrahim et al., 2020). Among these, sucrase catalyzes the hydrolysis of sucrose into glucose and fructose, thereby improving the transformation efficiency of soil organic matter (Wang et al., 2020). Amylase facilitates the decomposition of water-insoluble starch into soluble monosaccharides, increasing the labile carbon fraction in the soil (Su et al., 2019), of which the activity reflects the metabolic status of soil biomes and the conversion efficiency of soil carbon (Wang et al., 2020). Cellulase hydrolyzes cellulose into cellobiose and eventually glucose, serving as an indicator of straw degradation rate (Wickings et al., 2012). Several studies have demonstrated that variations in soil enzyme activity are closely correlated with changes in organic carbon fractions and can serve as early indicators for predicting trends in soil organic carbon speciation (Ma et al., 2014). Therefore, investigating the response mechanisms of soil carbon-converting enzymes, including sucrase, amylase, and cellulase, as well as carbon sequestering bacteria, to the process of carbon fraction changes in soil carbon fractions is essential for understanding the microbial and biochemical pathways involved in carbon cycling following the incorporation of corn straw and straw-derived biochar in black soil systems.

As the globally largest traditional agricultural country, China generates substantial crop straw resources. In 2022 alone, the major crops produced approximately 864,290,100 tons of straw, with corn straw accounting for approximately 288 million tons (33.36%) (Liu et al., 2024). The proportion of corn straw resources in China is extremely high and is mainly driven by corn production in Northeast China (Zhao et al., 2024). Currently, straw return is the most common method of straw resource utilization (Li et al., 2018). Incorporating straw not only enriches SOC inputs but also alters soil physicochemical properties and influences microbial community structure and enzymatic activities, thereby affecting SOC mineralization (Borase et al., 2020). In summary, the relationship between straw incorporation and soil SOC mineralization has received considerable attention. However, most existing studies have primarily focused on the associations between soil carbon fractions and SOC mineralization, while lacking a comprehensive understanding of the soil carbon transformation process after straw incorporation, particularly from an integration involving soil properties, carbon-converting enzymes, and microbial communities.

Additionally, while the relationship between straw incorporation and soil SOC mineralization has garnered significant attention, current research on the mechanisms of SOC mineralization under straw return conditions has predominantly focused on general soil bacteria and fungi, with limited investigation of functional microorganisms specifically involved in soil carbon metabolism. Particularly noteworthy is the *cbbL* gene, which encodes ribulose-1,5-bisphosphate carboxylase/oxygenase (RubisCO). As a key functional gene involved in microbe-mediated carbon fixation, it plays a central role in regulating the conversion of soil inorganic carbon into organic carbon and enhancing soil carbon sequestration function (Bu et al., 2023). The autotrophic microbial community represented by the *cbbL* gene fixes CO_2_ through the Calvin cycle pathway, significantly influencing the accumulation and stability of soil organic carbon, thereby serving as a critical link between soil carbon cycling and microbial functionality (Wang et al., 2024). Therefore, this study targeted degraded black soils in the agricultural regions of Northeast China by employing field experiments to assess the effects of different proportions of corn straw and straw-derived biochar on SOC mineralization. The analysis encompassed soil physicochemical properties, carbon fractions, carbon-converting enzyme activities, and structure of the *cbbL* bacterial community. The objective of this study was to elucidate the response of SOC mineralization to various straw management strategies, thereby contributing to a deeper understanding of carbon turnover and accumulation mechanisms in Northeast China’s agroecosystems. These insights are essential for optimizing straw resource utilization and developing effective carbon sequestration technologies. This study was conducted to (1) evaluate the changes in soil carbon fractions, enzyme activities, and *cbbL* bacterial community structure following straw and biochar incorporation; (2) determine the effects of straw and biochar return on SOC mineralization; and (3) assess the relative importance of abiotic factors in regulating SOC mineralization under different straw management practices.

## 2 Materials and methods

### 2.1 Soils, corn stover, and straw-derived biochar

The field experiment was conducted at the Experimental Station of Harbin Normal University, Heilongjiang Province, China (126°33′E, 45°51′N). The region has a typical semi-humid, temperate continental monsoon climate, with a mean annual temperature of −1 °C, an average frost-free period of 110 d, and an average annual precipitation of approximately 450 mm. The experimental site has been under continuous soybean-corn rotation for the past 17 years, and the soil type was classified as black soil. The topsoil (0–20 cm) had the following properties before the experiment: pH 5.42, total N 0.62 g·kg^−1^, available N 27.01 mg·kg^−1^, total P 0.75 g·kg^−1^, available P 105.66 mg·kg^−1^, total K 29.90 g·kg^−1^, and available K 121.20 mg·kg^−1^. The corn straw used in the experiment was collected from maize harvested in autumn of 2021 at the same site. After natural air-drying, the straw was cut into 1–5 cm segments with a carbon content of 40.3% on a dry weight basis. Straw-derived biochar was obtained from Liyang Activated Carbon Company and produced via the pyrolysis of corn straw under oxygen-limited conditions at 600 °C for 3 h, with a dry weight carbon content of 40.5%. Experimental design.

Based on the above-ground corn straw yield (6 t ha^−1^ dry weight) and its air-dried carbon concentration (40.3%), the total carbon input from full straw return was estimated at ≈ 2,500 kg C ha^−1^ (6,000 kg × 0.403). Using this value as the target carbon-equivalent input (2,500 kg C ha^−1^), five treatments were established according to a randomized complete block design. The treatments included the blank control with no carbon source (T0), corn straw applied alone (T1, 5.580 kg), straw-derived biochar applied alone (T2, 5.562 kg), combined application of corn straw and biochar at a 1:3 ratio (T3, 1.404 kg straw + 4.167 kg biochar), and combined application at a 3:1 ratio (T4, 4.194 kg straw + 1.386 kg biochar). Each treatment plot measured 9 m^2^ (3 m × 3 m), and a 1 m buffer zone was maintained around each plot to prevent cross-contamination.

Straw and straw-derived biochar were evenly applied to the soil surface in October 2021 and 2022, respectively, and were manually incorporated into the soil to a depth of approximately 20 cm. Prior to sowing, a one-time basal application of 338 g (equivalent to 375 kg·hm^−2^) of Red Square compound fertilizer (N: P_2_O_5_: K_2_O = 15:15:15) and 140 g (150 kg·hm^−2^) of urea was applied. No additional fertilizer was applied during the corn growing season. Corn (hybrid variety Xingdan 1, with a growth period of 122 d) was sown at the end of April 2023 and harvested in September. Standard field management practices were followed throughout the reproductive stage.

### 2.2 Soil sampling

Soil samples were collected in September 2023, after the corn harvest. In each plot, 15 sampling points are randomly selected, surface plant residues were removed, and intact soil cores were extracted from the 0–20 cm layer using an auger. Five random cores were composited into a single representative sample, and three soil samples were formed for each treatment. Seal all soil samples separately in sterile self-sealing bags and transported to the laboratory in dry ice containers. Upon arrival, visible crop root residue was removed. A portion of each sample was immediately sieved through a 1 mm mesh, placed into 50 mL sterile centrifuge tubes, and stored at −80 °C for subsequent DNA extraction and high-throughput sequencing. The remaining soil was air-dried and passed through 2 and 1 mm sieves, respectively, for the determination of soil physicochemical properties, carbon fractions, cumulative carbon mineralization, and carbon-converting enzyme activities. All analyses were performed in triplicate. Basic soil properties and corn yield data for each treatment are presented in Table 1 and Supplementary Table S1.

**Table 1:** The soil physicochemical properties of under different treatments.

Treatment	pH	TN (g·kg^−1^)	AN (mg·kg^−1^)	TP (g·kg^−1^)	AP (mg·kg^−1^)
T0	5.45 ± 0.02A	0.78 ± 0.01B	27.30 ± 0.01B	0.71 ± 0.04B	93.36 ± 1.31D
T1	5.40 ± 0.01B	0.25 ± 0.02E	26.60 ± 0.01B	0.75 ± 0.02AB	110.24 ± 0.10B
T2	5.40 ± 0.01B	1.05 ± 0.06A	25.20 ± 0.70C	0.76 ± 0.04AB	115.52 ± 3.13A
T3	5.39 ± 0.02B	0.61 ± 0.09C	28.23 ± 0.40A	0.78 ± 0.03A	97.07 ± 2.51C
T4	5.41 ± 0.01B	0.48 ± 0.09D	27.30 ± 0.70B	0.74 ± 0.01AB	112.09 ± 0.50AB

TN, total nitrogen; AN, alkaline dissolved nitrogen; TP, total phosphorus and AP, quick-acting phosphorus. The values are means ± SD (*n* = 3). Different letters indicate statistical differences at *p* < 0.05 between five treated soils. T0, the blank control with no carbon source; T1, corn straw applied alone; T2, corn straw-derived biochar applied alone; T3, combined application of corn straw and biochar at a 1:3 ratio; T4, combined application of corn straw and biochar at a 3:1 ratio.

### 2.3 Soil organic carbon fractions

The soil carbon fractions were determined using conventional chemical methods (Mao et al., 2025). Briefly, the soil OC content was measured via potassium dichromate oxidation with concentrated sulfuric acid. Dissolved organic carbon (DOC) was extracted with distilled water and quantified by external heating with potassium dichromate. Labile organic carbon (LOC) was assessed by oxidation with 333 mmol·L^−1^ KMnO_4_. The microbial biomass carbon (MBC) was determined using the chloroform fumigation–extraction method.

### 2.4 Soil C mineralization

Cumulative SOC mineralization was determined following the procedure described by Shu et al. (2023). Briefly, 10 g of fresh soil was placed in a 25 mL glass beaker, and another 25 mL beaker containing 15 mL of 1 mol·L^−1^ NaOH solution was positioned adjacent to it inside a sealed 250 mL plastic incubation jar. The jars were incubated at 25 °C in a constant-temperature chamber for 15 d. During incubation, CO_2_ released from microbial decomposition was absorbed by the NaOH solution. After incubation, the residual NaOH was quantified by titration with 0.1 mol·L^−1^ HCl, and the amount of CO_2_ evolved was used to quantify the cumulative SOC mineralization.

Equation 1 was used to compute the carbon mineralization efficiency:


(1) $$\begin{array}{l} \mathrm{CME}(\mathrm{mg}\;{\mathrm{CO}}_{2}-C\cdot g^{-1}\;\mathrm{SOC})=\text{Cumulative}\;\mathrm{SOC}\;\\ \text{mineralization}\;(\mathrm{mg}\;{\mathrm{CO}}_{2}-C\cdot {\mathrm{kg}}^{-1})/\mathrm{SOC}(g\cdot {\mathrm{kg}}^{-1}) \end{array}$$

where CME indicates the carbon mineralization efficiency, and SOC indicates the soil organic carbon content.

### 2.5 Soil carbon-converting enzymes activity

The activities of soil cellulase (SCL), amylase (SAI), and sucrase (SSC) were determined using the 3,5-dinitrosalicylic acid (DNS) colorimetric method, following the procedure described by Guan, 1980. Briefly, 2 g of air-dried soil was weighed and mixed with 1 mL of toluene, 10 mL of acetate buffer (pH 5.0), and 10 mL of the corresponding reaction substrate: 1.0% sodium carboxymethyl cellulose solution for cellulase, 1.0% soluble starch solution for amylase, and 0.35 mol·L^−1^ sucrose solution for sucrase. The mixtures were incubated in a shaking water bath at 37 °C for the respective reaction periods followed by filtration. A 2 mL aliquot of the filtrate was then mixed with 3 mL of DNS reagent, and the mixture was heated in a boiling water bath for 30 min to allow full color development. Absorbance was measured at 508 nm using a spectrophotometer. Enzyme activities were expressed as the amount of glucose released per gram of soil after incubation for 72 h for cellulase, 48 h for amylase, and 24 h for sucrase.

### 2.6 Soil DNA extraction and Illumina-MiSeq high-throughput sequencing

Genomic DNA was extracted from fresh soil (0.5 g) using the PowerSoil^®^ DNA Isolation Kit (MoBio Laboratories, Carlsbad, CA, United States), following the manufacturer’s protocol. DNA purity and integrity were assessed by 0.8% agarose gel electrophoresis. The carbon fixation gene *cbbL* was amplified using primers *cbbL*F (5′-GACTTCACCAAAGACGACGA-3′) and *cbbL*R (5′-TCGAACTTGATTTCTTTCCA-3′). PCR amplification was performed under the following conditions: initial denaturation at 95 °C for 5 min, followed by 28 cycles of denaturation at 95 °C for 45 s, annealing at 55 °C for 50 s, and extension at 72 °C for 45 s, and a final extension at 72 °C for 10 min. Each sample was amplified in triplicate. The PCR products were purified using the AxyPrep DNA Gel Recovery Kit (Axygen, United States), and their concentrations were evaluated using 2% agarose gel electrophoresis. Qualified amplicons were sequenced on an Illumina MiSeq PE300 platform (Illumina Inc., San Diego, CA, United States) at Beijing Allwegene Technology Co., Ltd. Image analysis, base calling, and error estimation were conducted using the Illumina MiSeq Analysis Pipeline.

Raw sequencing data were subjected to quality control for sequence recognition, including chimera detection and removal, to obtain high-quality valid sequences. These sequences were clustered into operational taxonomic units (OTUs) at a 97% similarity threshold using the Usearch platform (v2.7.1), and an OTU abundance matrix was constructed for each sample. OTUs shared among the samples were identified based on the OTU matrix. Microbial community richness and evenness were assessed using Chao1, Observed species, and Shannon diversity indices. Taxonomic classification of OTUs was performed using QIIME (v1.8.0) against the SILVA reference database (Release 128/132), and species-level annotation was conducted at the phylum, class, and genus levels.

### 2.7 Statistical analysis

The data were compiled and organized using Microsoft Excel 2017, and all graphical values represent the mean values. Analysis of variance (ANOVA) and post-hoc Duncan’s multiple range tests (*p* < 0.05) were conducted to evaluate the differences in soil properties, carbon fractions (OC, DOC, LOC, and MBC), SOC mineralization, and carbon-converting enzyme activities (SCL, SAI, and SSC). Statistical analyses and visualizations, including bar charts and stacked histograms, were performed using Origin 2022 to illustrate differences in SOC mineralization, enzyme activity, microbial taxonomic composition, and community abundance. Principal coordinate analysis (PCoA) was used to assess similarities in the *cbbL* bacterial community structures across treatments. Redundancy analysis (RDA) was used to identify the soil environmental factors influencing *cbbL* community composition. Mantel tests were used to evaluate the correlations between *cbbL* bacterial communities and soil environmental parameters. The structural equation modeling (SEM) was employed to determine the direct and indirect effects of soil variables on SOC mineralization. The multivariate analyses (PCoA, RDA, Mantel, and SEM) were conducted using R (v4.3.3).

## 3 Results and analysis

### 3.1 Soil carbon fractions

Table 2 showed the changes in soil carbon fractions under different treatments. Compared to T0, the application of corn straw and straw-derived biochar at different ratios significantly affected the soil OC, DOC, and MBC contents (*p* < 0.05), while no significant differences were observed in the LOC content across treatments (Table 2). Specifically, the soil OC content of the T3 and T4 treatments increased significantly by 29.43 and 20.62%, respectively. The soil DOC content of the T4 treatment increased significantly by only 64.12%, whereas the other treatments resulted in a significant decrease. The soil MBC content of the T1 treatment decreased significantly by 14.73%, but increased significantly in all other treatments (Table 2).

**Table 2:** Changes in soil carbon fractions under different treatments.

Treatment	OC (g·kg^−1^)	DOC (mg·kg^−1^)	LOC (g·kg^−1^)	MBC (mg·kg^−1^)	Cumulative C mineralization (mg CO_2_-C·kg^−1^)	CME (mg CO_2_-C·g^−1^ SOC)
T0	11.69 ± 0.69C	312.19 ± 30.54B	2.65 ± 0.22A	150.88 ± 1.19D	899.27 ± 74.21B	77.18 ± 9.35AB
T1	12.61 ± 1.05 BC	240.93 ± 13.57C	2.89 ± 0.13A	128.66 ± 0.36E	1019.62 ± 72.02A	80.98 ± 4.32A
T2	11.46 ± 0.40C	139.13 ± 6.79D	2.78 ± 0.18A	171.58 ± 0.50C	990.00 ± 18.35AB	86.55 ± 8.45A
T3	15.13 ± 1.38A	237.53 ± 16.97C	2.65 ± 0.25A	221.21 ± 0.48A	1018.68 ± 61.03A	67.73 ± 6.94B
T4	14.10 ± 0.34AB	512.39 ± 13.57A	2.71 ± 0.05A	193.83 ± 1.37B	940.64 ± 22.03AB	66.71 ± 0.92B

Different capital letters indicate significant differences at *p* < 0.05 among treatments, based on the Duncan’s significance difference test.

Compared to T0, the cumulative carbon mineralization in the soils of the T1 and T3 treatments increased significantly by 13.38 and 13.28%, respectively (*p* < 0.05; Table 2), whereas no significant differences were observed for T2 and T4 (Table 2). Furthermore, the application of corn straw and straw-derived biochar at different ratios did not significantly affect the soil CME (Table 2).

### 3.2 Soil carbon-converting activities

**Figure 1:**
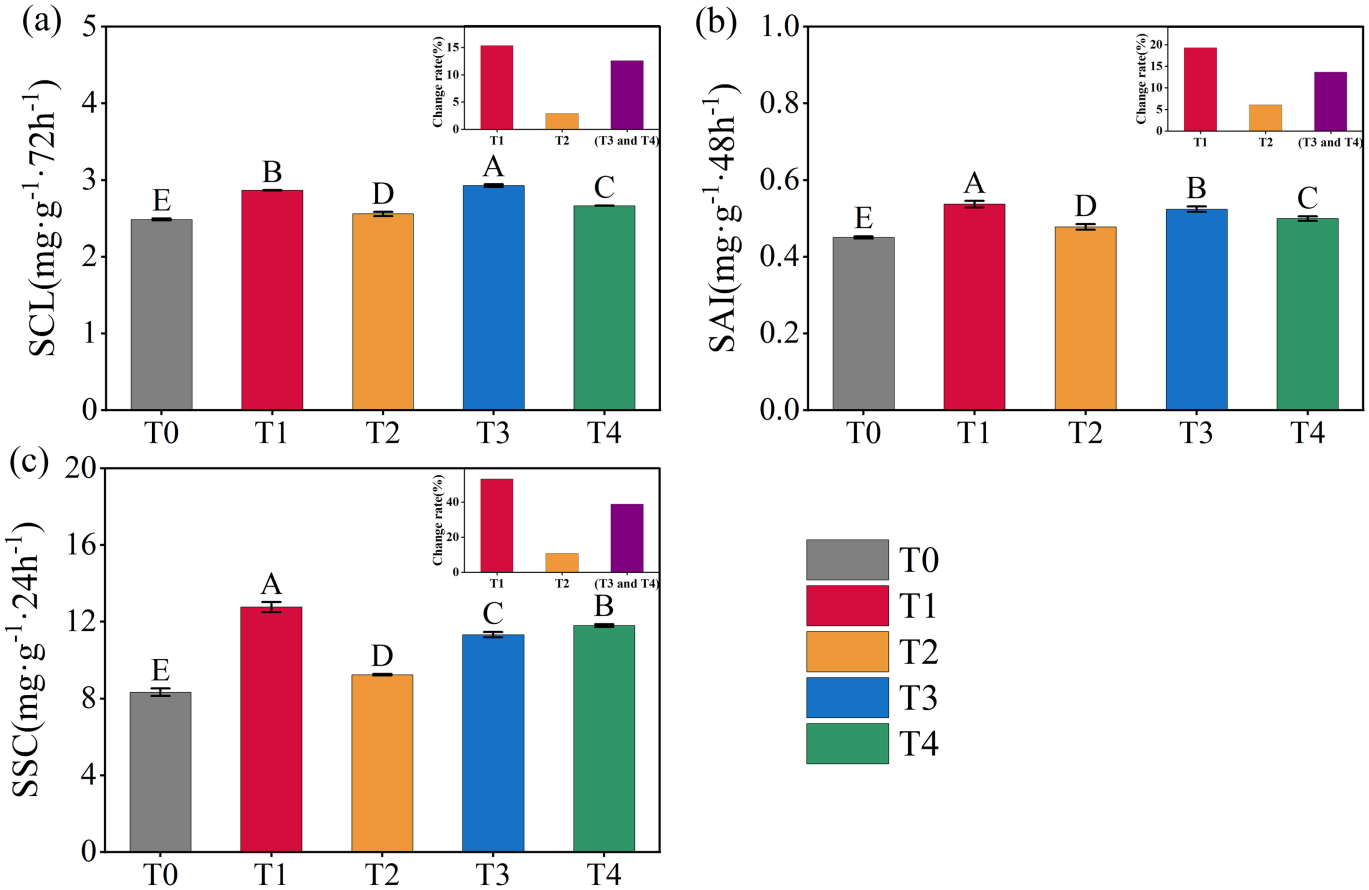
Changes of soil carbon-converting enzyme activities under different treatments. SCL **(a)**, SAI **(b)**, and SSC **(c)**. Error bars indicate standard deviation. Different capital letters indicate significant differences at *p* < 0.05 among treatments, based on the Duncan’s significance difference test.

Variations of soil carbon-converting enzyme activities under different treatments were presented in Figure 1. Compared to T0, all treatments involving the application of corn straw and straw-derived biochar significantly enhanced the activities of soil SCL, SAI, and SSC (*p* < 0.05, Figure 1). Among the treatments, T3 resulted in the highest increment rate in SCL activity (17.84%), whereas T1 exhibited the greatest increment rate in SAI and SSC activities (19.26 and 53.18%). Overall, the order of enzyme activity improvement across the four straw return treatments followed the pattern: corn straw > combined straw + straw-derived biochar > straw-derived biochar (Figure 1).

### 3.3 Soil *cbbL* bacterial community structure

**Figure 2:**
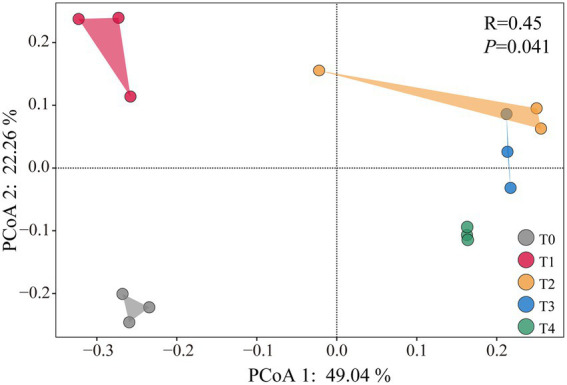
Principal component analysis of soil *cbbL* bacterial community diversity under application of different proportions of corn stover and straw-derived biochar.

Principal component analysis of soil *cbbL* bacterial community diversity under different treatments can be observed in Figure 2. Compared to T0, only the T1 treatment significantly increased the Shannon and Simpson diversity indices (*p* < 0.05), whereas no significant changes were observed under other treatments (Supplementary Figure S1). PCoA 1 and PCoA 2 explained 49.04 and 22.26% of the total variation, respectively (Figure 2). The bacterial community structures in all straw and biochar treatments were significantly separated from those of the control (*p* < 0.05). Among them, T1 exhibited significant separation from the other treatments (*p* < 0.05), whereas T2 and T3 showed only partial separation (Figure 2).

**Figure 3:**
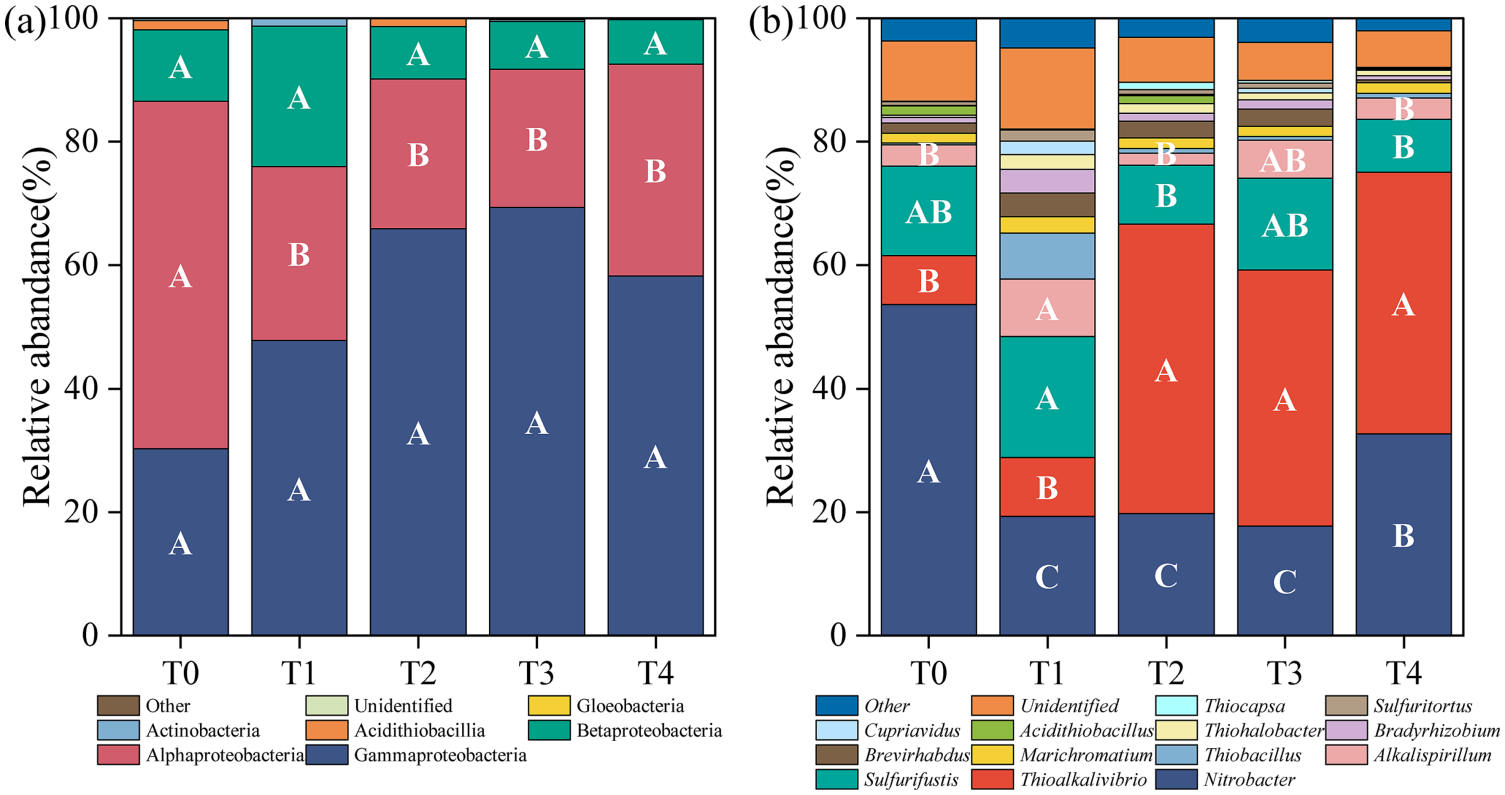
Composition and relative abundance of soil *cbbL* bacterial community under different treatments. The class levels **(a)** and the genus levels **(b)**.

Figure 3 listed the composition and relative abundance of soil *cbbL* bacterial community under different treatments. A total of 5 phyla, 10 classes, and 64 genera were identified from the annotated OTU sequences. At the phylum level, Proteobacteria overwhelmingly dominated all treatments, accounting for 99.09–99.69% of the community. Within Proteobacteria, the dominant classes included Gammaproteobacteria (30–69%), Alphaproteobacteria (22–56%), and Betaproteobacteria (7–23%) (Figure 3A), with the relative abundance of Alphaproteobacteria significantly reduced in all treatments compared to the control (*p* < 0.05; Figure 3A). At the genus level, the dominant bacterial taxa (relative abundance >5%) across all treatments were *Nitrobacter* (18–54%), *Thioalkalivibrio* (8–47%), and *Sulfurifustis* (9–20%) (Figure 3B). Compared to T0, *Alkalispirillum* emerged as the dominant genus in T1 and T3, with relative abundances of 9 and 6%, respectively, whereas *Thiobacillus* was predominant in T1 (7%). The relative abundance of *Nitrobacter* declined significantly following the application of various proportions of corn stover and stover-derived biochar (*p* < 0.05). In contrast, the relative abundance of *Thioalkalivibrio* significantly increased in T2, T3, and T4 (*p* < 0.05), and that of *Sulfurifustis* significantly increased in T1 but declined in T2 and T4 (*p* < 0.05, Figure 3B).

### 3.4 Comprehensive analysis of soil physicochemical properties, carbon fractions, carbon-converting enzymes, and *cbbL* bacterial community structure

#### 3.4.1 Correlation analysis

**Figure 4:**
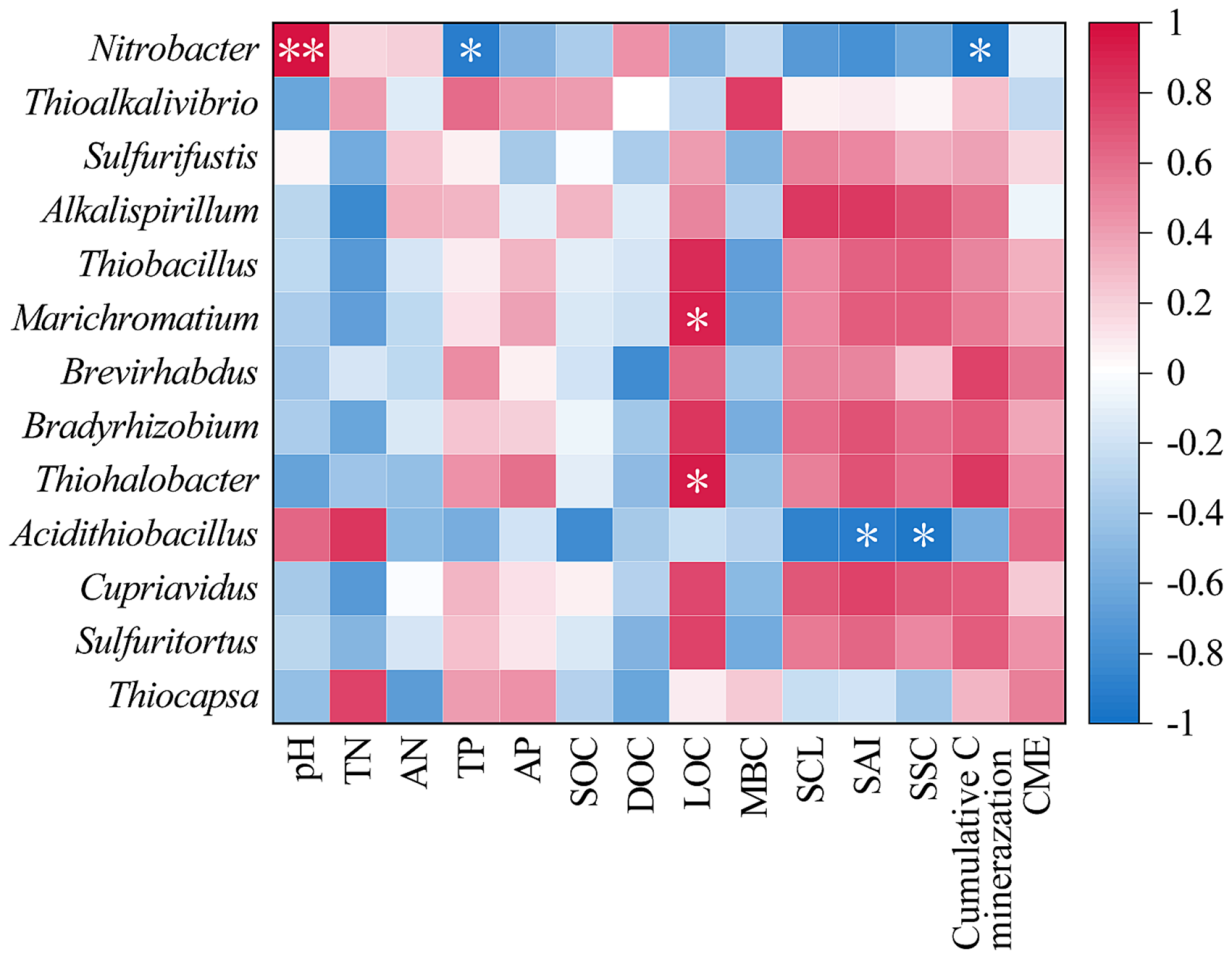
The correlation heat map of soil physicochemical properties, carbon fractions and carbon-converting enzyme activities with soil *cbbL* bacterial community. *p < 0.05.

The correlation heatmap of soil properties, carbon components, and carbon invertase activity with the *cbbL* bacterial community structure shown in Figure 4. It demonstrated that *Nitrobacter* was significantly positively correlated with pH (*p* < 0.01) and significantly negatively correlated with the TP content and cumulative soil carbon mineralization (*p* < 0.05; Figure 4). *Marichromatium* and *Thiohalobacter* exhibited significant positive correlations with the LOC content (*p* < 0.05; Figure 4). Additionally, *Acidithiobacillus* was positively correlated with SAI and SSC activities (*p* < 0.05; Figure 4).

**Figure 5:**
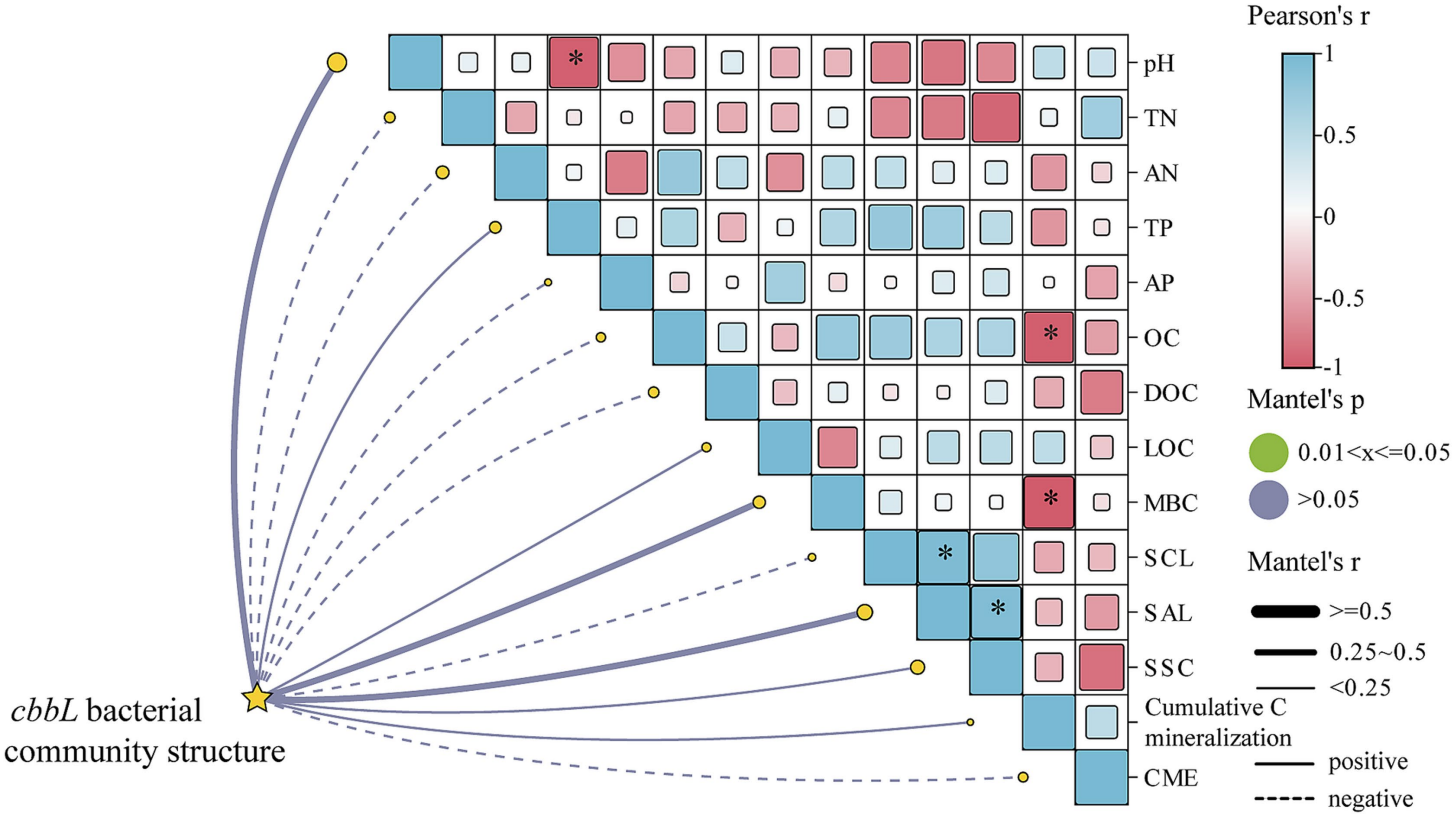
Metal test heatmap of soil physicochemical properties, carbon composition and carbon-convertase activity with *cbbL* bacterial community. *p < 0.05.

The correlation heatmap of soil properties, carbon components, and *cbbL* bacterial community diversity was presented in Figure 5. It indicated that the Observed species index was significantly positively correlated with MBC content (*p* < 0.05), while the Shannon index was significantly negatively correlated with DOC content (*p* < 0.05). Additionally, the Simpson index demonstrated a significant negative correlation with MBC content (*p* < 0.05; Supplementary Figure S2). The Mantel test further indicated that soil OC and MBC were significantly negatively correlated with cumulative soil carbon mineralization (*p* < 0.05). Soil pH was significantly negatively correlated with the TP content (*p* < 0.05). Moreover, significant positive correlations were observed between SCL and SAI activities and between SCL and SAI with SSC activity (*p* < 0.05; Figure 5).

#### 3.4.2 Structural equation model analysis

**Figure 6:**
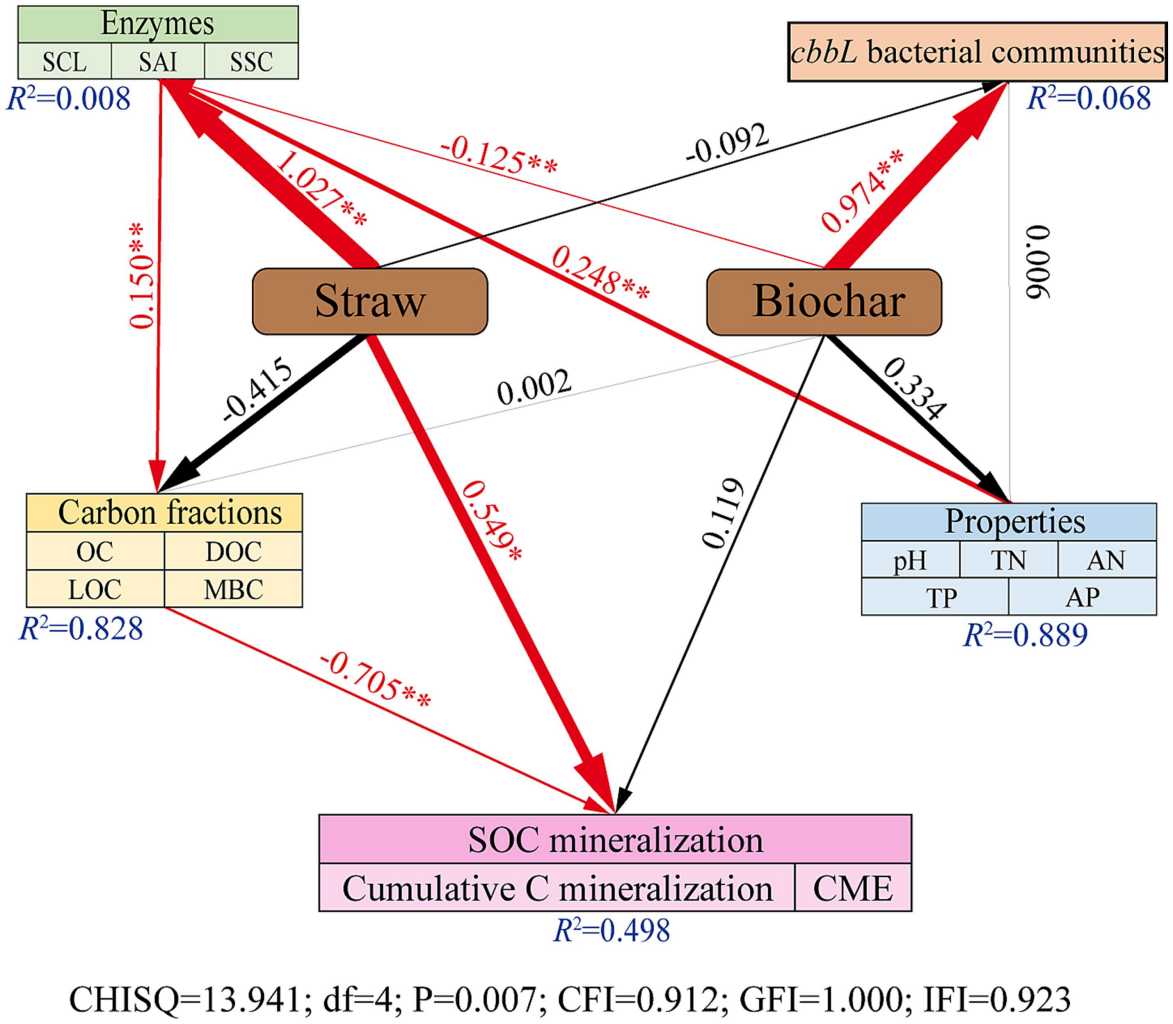
Structural equivalence model (SEM) analyze the direct and indirect effects of SOC mineralization on the application of corn straw and straw-derived biochar. Red solid arrows indicate significant paths. Black arrows represent tested, but not significant paths. The arrow width is proportional to the strength of the relationship. Goodness-of fit statistics for the model are shown below the model. *p < 0.05, **p < 0.01.

Figure 6 presented the result of Structural Equation Modeling (SEM) analysis. SEM revealed that the application of corn straw exerted a significant positive influence on soil carbon-converting enzyme activities (*p* < 0.01) and on the SOC mineralization (*p* < 0.05; Figure 6). In contrast, straw-derived biochar significantly enhanced the structure of the *cbbL* bacterial community (*p* < 0.01) but negatively affected the activity of carbon-converting enzymes (*p* < 0.01; Figure 6). Additionally, soil physicochemical properties had a significant positive effect on enzyme activities (*p* < 0.01), which positively influenced soil carbon components (*p* < 0.01). Notably, the accumulation of soil carbon components exhibited a significant negative effect on SOC mineralization (*p* < 0.01), indicating a suppressive feedback mechanism within the soil carbon cycle (Figure 6).

## 4 Discussion

### 4.1 Effects of different proportions of corn straw and straw-derived biochar on soil carbon fractions

In the context of soil fertility assessment, although active organic carbon fractions, such as LOC, DOC, and MBC, comprise only a small proportion of total soil OC, they are more sensitive indicators of OC dynamics (Yu et al., 2007). LOC demonstrates a high sensitivity to environmental fluctuations and is closely associated with soil functional properties (Zhang et al., 2014b). DOC serves as a mobile carbon source and an essential energy source for microbial activity, playing a pivotal role in biogeochemical cycling of soil carbon (Cressey et al., 2018). MBC represents the most dynamic component of soil organic matter, reflecting both microbial abundance and metabolic potential (Moura et al., 2018). Collectively, variations in these fractions indicate changes in the stability and turnover of soil OC (Shu et al., 2023). Straw return practices generally improve soil structure and promote carbon sequestration, thereby altering the composition of soil organic carbon fractions (Six et al., 2000). In this study, although the application of corn straw or straw-derived biochar alone did not significantly influence the soil OC content, their co-application resulted in a substantial increase (Table 2), which partially diverges from previous findings, likely because of the differences in straw type, incorporation timing, and soil characteristics (Six et al., 2000; Cressey et al., 2018; Moura et al., 2018; Shu et al., 2023; ElDesouki et al., 2024; Li et al., 2025; Yuan et al., 2025). In terms of active soil organic carbon fractions, our results indicated that the application of varying proportions of corn straw and straw-derived biochar did not significantly affect the soil LOC content in the degraded black soil area. However, their co-application significantly increased DOC and MBC levels, except in the treatment with a 1:3 corn straw to biochar ratio (Table 2). These findings suggested that the co-application provided a more bioavailable and readily utilizable carbon source, thereby enhancing microbial biomass. This increase in microbial activity may contribute to the observed increase in total soil organic carbon following the combined application of corn straw and straw-derived biochar.

In this study, the cumulative mineralization of SOC significantly increased following the application of corn straw, whereas no significant changes were observed with the straw-derived biochar treatment (Table 2). Agronomic performance indicators, including plant height, stem thickness, thousand-grain weight, fresh yield, and dry yield, were significantly enhanced under corn straw application compared to other straw return treatments (Supplementary Table S1). This may be attributed to the fact that under equal carbon input conditions, corn straw can promote rapid SOC mineralization, thereby accelerating soil carbon cycling and making nutrients more readily available for plant growth and development (Wang C. Y. et al., 2023). In contrast, straw-derived biochar appears to be more effective at promoting long-term carbon sequestration in soils (Vineet et al., 2024). Furthermore, SEM confirmed that corn straw exerted a direct and positive effect on SOC mineralization (Figure 6), which is consistent with the observed increase in mineralization following straw addition and the lack of significant changes under biochar treatment.

### 4.2 Effects of application of different proportions of corn straw and straw-derived biochar on soil carbon-converting enzymes and *cbbL* bacterial community structure

Numerous studies have demonstrated that enzymes function as sensitive indicators of microbial activity and serve as critical intermediaries that link microbial metabolism to soil carbon cycling (Ashraf et al., 2021; Hu et al., 2023). In this study, the application of corn straw and straw-derived biochar, whether applied individually or in combination, significantly enhanced the activity of three key carbon-converting enzymes: soil SCL, SAI, and SSC (Figure 1). This finding is consistent with previous research showing that straw incorporation can promote enzyme activity, likely because of the external carbon sources provided by straw and biochar, which stimulate enzymatic reactions by increasing available substrates and offering additional binding sites for enzyme activity (Jiao et al., 2015; Bo et al., 2017). Moreover, the enhancement of enzyme activity followed a consistent trend: the highest under corn straw application, followed by the co-application of straw and biochar, and the lowest under the biochar-only treatment (Figure 1). This represents a novel finding in this study. A possible explanation could be that compared with straw-derived biochar rich in recalcitrant aromatic carbon, corn straw contained more labile carbon forms, such as polysaccharides, which were more readily decomposed by microorganisms. This rapid degradation supplies a greater abundance of substrates for soil carbon-converting enzymes, thereby facilitating enhanced carbon transformation processes (Li et al., 2023; Sun and Han, 2024). Therefore, we speculated that under equal carbon input, a higher proportion of corn straw in the applied carbon source could lead to enhanced activity of soil carbon-transforming enzymes. This hypothesis is supported by the SEM established in this study, which revealed that corn straw application exerted a direct positive effect on enzyme activity, whereas straw-derived biochar application had a direct negative effect (Figure 6).

The soil *cbbL* bacterial community is a key driver of soil carbon cycling and plays a vital role in carbon uptake and utilization by crops, whereas its diversity is essential for maintaining soil health and quality (Yuan et al., 2012a; Qin et al., 2021). In this study, all straw return treatments significantly altered the composition of the *cbbL* bacterial community, with a notable decrease in the relative abundance of the dominant class Alphaproteobacteria and genus *Nitrobacter* (Figure 3). These findings are consistent with those of previous studies indicating that both corn straw and straw-derived biochar can reshape soil microbial communities (Yuan et al., 2012b; Wang X. J. et al., 2023). At the same time, we found that soil pH was significantly positively correlated with the relative abundance of *Nitrobacter* (Figure 4). This may be due to the fact that soil *Nitrobacter* is greatly affected by pH changes, and the application of straw and straw-derived biochar can lead to soil acidification, which may reduce their ability to compete with other bacterial taxa, which in turn shows a decrease in the relative abundance of soil *Nitrobacter*. Furthermore, the bacterial community structures following the application of biochar alone or in combination with straw were more similar, and both differed significantly from the community structure observed under straw-only treatment (Figure 2). Notably, the relative abundance of Thiobacillus significantly increased under biochar treatments, emerging as a new dominant genus (Figure 3). These results indicate that different carbon sources have different effects on soil microorganisms, and straw-derived biochar may have a more profound effect on the composition and abundance of soil *cbbL* bacterial communities than corn straw. This may be because biochar has high porosity, large specific surface area, and rich functional groups, and its application to soil can provide a stable microenvironment for autotrophs and enhance their attachment and metabolic activity, resulting in different compositions and concentrations of soil *cbbL* bacterial degradable substrates (Weng et al., 2017). This speculation was further supported by the SEM results, which indicated that biochar application had a direct positive effect on the diversity of the *cbbL* bacterial community, while corn straw did not significantly affect the microbial diversity (Figure 6). In addition, we confirmed that the application of biochar had a significant positive effect on the change of soil carbon-converting enzymes activity (Figure 6). In the correlation heat map, it was found that the dominant genus *Acidithiobacillus* was significantly correlated with soil SAI and SSC activities (Figure 4). Therefore, we speculate that the application of biochar to soil may provide a more suitable environment for soil microorganisms through the physical characteristics of biochar itself, and these changes potentially change the functional genes of soil carbon sequestration, which in turn affects the enzymes related to the soil carbon cycle, and significantly affects the carbon fixation pathway of soil microorganisms and the bacterial community structure of soil carbon sequestration.

### 4.3 Impact of soil carbon content, *cbbL* bacterial community structure, and carbon transformation enzyme activity on soil organic carbon mineralization

The process of soil carbon mineralization can be regulated by multiple environmental factors (He et al., 2024). Our SEM results demonstrated that both corn straw and straw-derived biochar application significantly influenced the activity of soil carbon-converting enzymes (Figure 6). These enzyme activities directly and positively affected the soil carbon component levels, which significantly drove SOC mineralization. This finding contrasts with those of previous studies, where organic carbon mineralization following exogenous carbon input was primarily mediated by microbial communities (He et al., 2024; Ren et al., 2024). In the degraded black soil region examined in this study, the short-term incorporation of straw and biochar likely increased the availability of substrates for carbon-converting enzymes, thereby triggering enzymatic responses that subsequently altered the carbon fractions and promoted carbon mineralization. VPA identified soil carbon-converting enzymes as the dominant contributors to soil carbon mineralization (Supplementary Figure S3). Additionally, a significant negative correlation between soil OC and MBC contents and cumulative carbon mineralization (Figure 5) further validated the SEM findings. Notably, neither the SEM nor the Mantel test revealed a significant association between the structure of the soil *cbbL* bacterial community and organic carbon mineralization (Figures 5, 6). This diverged from prior research (Wang et al., 2025), suggesting that in this specific degraded black soil system in Northeast China, shifts in soil carbon mineralization may not be driven by microbial carbon fixation pathways such as CO_2_ or CH_4_ assimilation. Furthermore, we observed that the composition and diversity of the *cbbL* bacterial community were significantly positively correlated with LOC, MBC, TN, and TP (Figure 4; Supplementary Figure S2). Soils with a higher microbial diversity can support more robust microbial functions, thereby enhancing nutrient cycling (Louca et al., 2018; Maron et al., 2018), which may further explain the patterns observed in this study. Although this study preliminarily revealed the effects of different ratios of corn straw and straw biochar on soil carbon conversion, the priming effect (PE) triggered by exogenous carbon input has not been fully evaluated. However, the direction and amplitude of PE and its microbial driving mechanism are key prerequisites for accurately predicting the long-term carbon sequestration potential of soil. Unfortunately, under the field test scale, the PE induced by straw returning shows high spatiotemporal variability, unpredictability and strong environmental dependence, which is regulated by the coupling of multiple factors such as straw chemical composition, soil background attributes, climate fluctuation and timing of returning, which makes it difficult to systematically deduce the existing results to the complex and diverse straw returning scenarios in the black soil region of China, which limits the in-depth analysis and regional scale simulation of its carbon sequestration mechanism.

## 5 Conclusion

Although the effects of varying proportions of corn straw and straw-derived biochar on SOC, DOC, and MBC differed, all treatments involving their combined application resulted in significant increases in these carbon components. Cumulative SOC mineralization increased significantly only when corn straw was applied alone or in combination with straw-derived biochar at a 1:3 ratio. The differentiation and structural changes in the soil *cbbL* bacterial community were primarily driven by the application of straw-derived biochar, whereas the enhancement of soil carbon-converting enzyme activity was driven by both corn straw and its derived biochar. Moreover, the co-application of these two carbon sources directly altered soil carbon composition and SOC mineralization by stimulating carbon-converting enzyme activity. Collectively, these findings suggest that corn straw and straw-derived biochar amendments adopt distinct strategies to improve soil carbon composition, yet both enhance soil quality and the carbon pool. Nevertheless, after comprehensively accounting for the energy inputs and economic costs of biochar production, full-rate direct straw return retains a comparative advantage in simultaneously increasing crop yield and soil quality.

## Data Availability

The original contributions presented in the study are publicly available. This data can be found here: Genome Sequence Archive (GSA) under accession number CRA031120.
