# Supplementary material for: Impact of corn straw and straw-derived biochar returning to the field on soil carbon fractions, carbon-converting enzyme activities, and cbbL bacterial community structure
Source: Front Microbiol. 2025 Nov 3;16:1611691. doi: 10.3389/fmicb.2025.1611691 (PMC12620358; doi:10.3389/fmicb.2025.1611691)
Supplement: Supplementary file 1 [file Table_1.docx]

Table S1. The Corn yield indexes of under different treatments.

| **Treatment** | T0 | T1 | T2 | T3 | T4 |
| --- | --- | --- | --- | --- | --- |
| **Plant height (cm)** | 195.40E | 224.61A | 199.22D | 204.63C | 217.80B |
| **Stem thickness (mm)** | 58.18E | 66.85A | 58.90D | 63.55C | 65.14B |
| **1000-grain weight (g)** | 323.46E | 361.19A | 342.79D | 356.18C | 358.01B |
| **Fresh yield (kg·hm^-2^)** | 7 188E | 12 842A | 8 760D | 10 685C | 11 934B |
| **Dry yield (kg·hm^-2^)** | 4 106E | 7 695A | 5 238D | 6 325C | 7 180B |

Different letters indicate statistical differences at *p*<0.05 between five treated soils.
