# Supplementary material for: Impact of corn straw and straw-derived biochar returning to the field on soil carbon fractions, carbon-converting enzyme activities, and cbbL bacterial community structure
Source: Front Microbiol. 2025 Nov 3;16:1611691. doi: 10.3389/fmicb.2025.1611691 (PMC12620358; doi:10.3389/fmicb.2025.1611691)
Supplement: Supplementary file 2 [file Data_Sheet_1.docx]

Supplementary Material

## Supplementary Figures

**
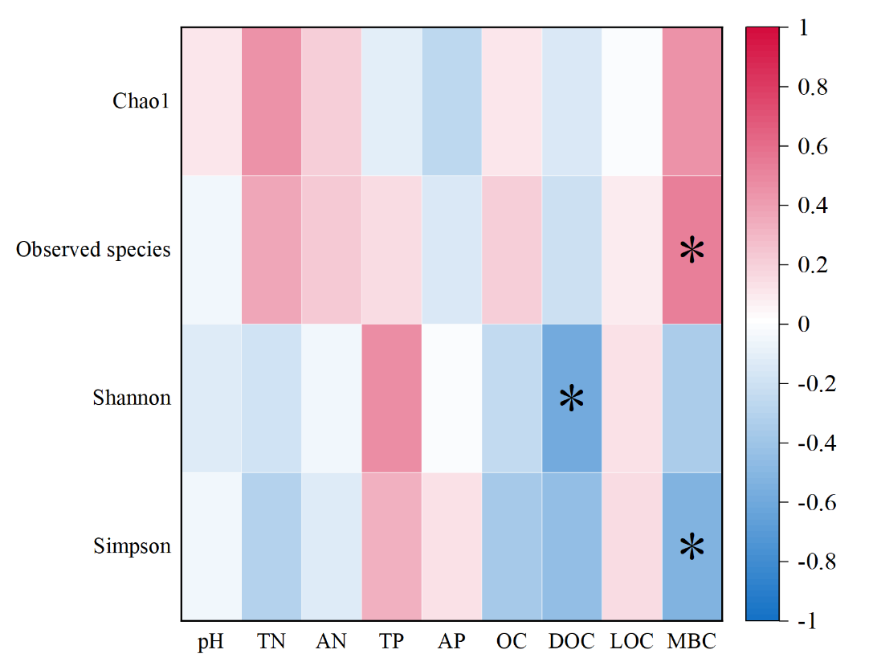
**
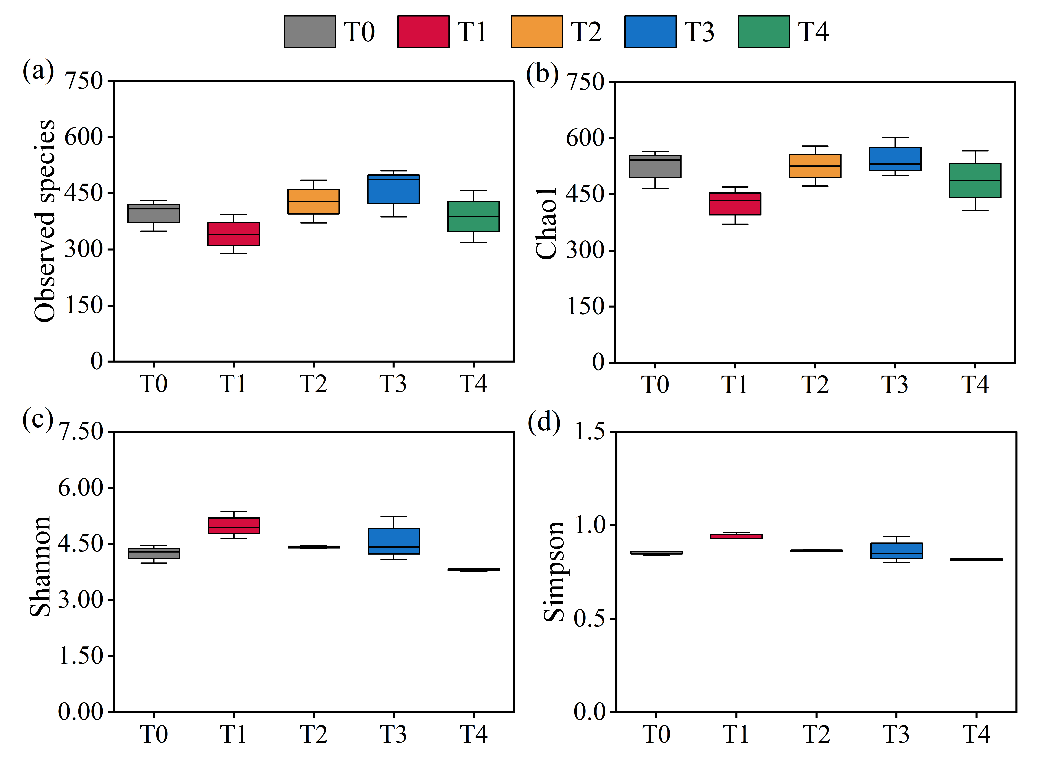
**Supplementary Figure 1.** Change in the *α*-diversity of soil *cbbL* bacterial community under different treatments.

**Supplementary Figure 2.** Correlation heat map of soil physicochemical properties, carbon content and α-diversity of soil *cbbL* bacterial community


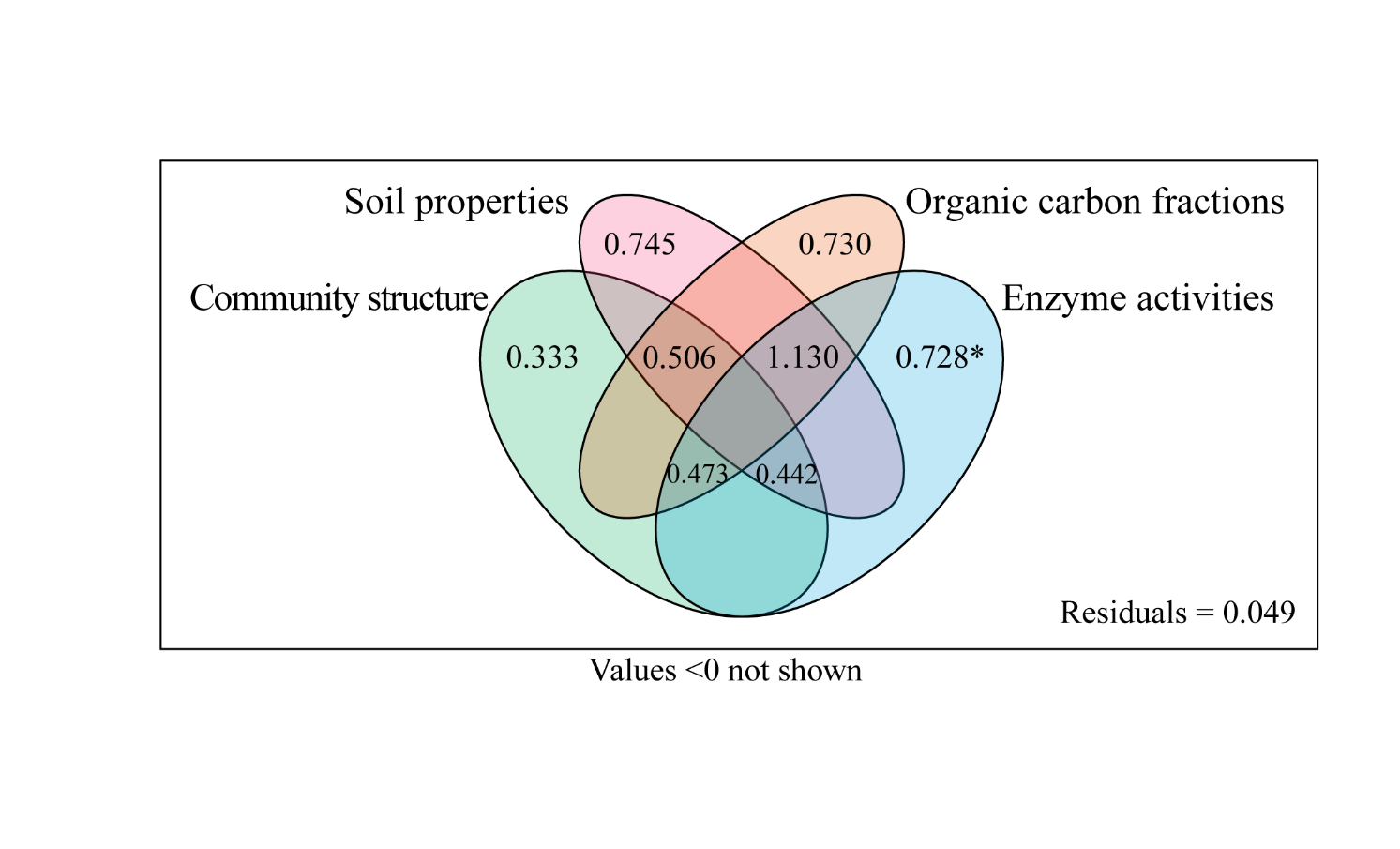
 **Supplementary Figure 3.** Variance decomposition analysis (VPA) of the relative contribution of soil physicochemical properties, carbon fractions, carbon-converting enzyme activities, and *cbbL* bacterial community to the variation of SOC mineralization.
